# A high-fat diet stimulates fibroblast growth factor 23 formation in mice through TNFα upregulation

**DOI:** 10.1038/s41387-018-0037-x

**Published:** 2018-05-29

**Authors:** Philipp Glosse, Abul Fajol, Frank Hirche, Martina Feger, Jakob Voelkl, Florian Lang, Gabriele I. Stangl, Michael Föller

**Affiliations:** 1 Institute of Agricultural and Nutritional Sciences, Martin Luther University Halle-Wittenberg, 06120 Halle (Saale), Germany; 2 Department of Physiology and Molecular Biology, Bangladesh University of Health Sciences (BUHS), Dhaka, 1216 Bangladesh; 3 Department of Physiology, University of Tübingen, 72076 Tübingen, Germany

## Abstract

**Background/objectives:**

Bone-derived fibroblast growth factor 23 (FGF23) is a hormone that suppresses renal phosphate reabsorption and calcitriol (i.e., 1,25(OH)_2_D_3_) formation together with its co-receptor Klotho. FGF23- or Klotho-deficient mice suffer from rapid aging with multiple age-associated diseases, at least in part due to massive calcification. FGF23 is considered as a disease biomarker since elevated plasma levels are observed early in patients with acute and chronic disorders including renal, cardiovascular, inflammatory, and metabolic diseases. An energy-dense diet, which induces sequelae of the metabolic syndrome in humans and mice at least in part by enhancing pro-inflammatory TNFα formation, has recently been demonstrated to stimulate FGF23 production.

**Methods:**

We investigated the relevance of TNFα for high-fat diet (HFD)-induced FGF23 formation in wild-type (tnf^+/+^) and TNFα-deficient (tnf^−/−^) mice.

**Results:**

Within 3 weeks, HFD feeding resulted in a strong increase in the serum FGF23 level in tnf^+/+^ mice. Moreover, it caused low-grade inflammation as evident from a surge in hepatic *Tnfα* transcript levels. TNFα stimulated *Fgf23* transcription in UMR106 osteoblast-like cells. Serum FGF23 was significantly lower in tnf^−/−^ mice compared to tnf^+/+^ mice following HFD. Serum phosphate and calcitriol were not significantly affected by genotype or diet.

**Conclusions:**

We show that HFD feeding is a powerful stimulator of murine FGF23 production through TNFα formation.

## Introduction

The hormone fibroblast growth factor 23 (FGF23) is mainly produced by osteoblasts and osteocytes in the bone^1^. Its renal effects include inhibition of phosphate reabsorption and calcitriol formation^1, 2^. Calcitriol is the biologically active form of vitamin D. The renal effects of FGF23 are mediated by a receptor which requires the protein α-Klotho (referred to as Klotho in the following) as an obligatory co-receptor^1^.

Klotho was originally discovered in 1997 as an anti-aging protein^3–5^. Klotho-deficient mice have an extremely short life span of a few weeks only and exhibit many disorders associated with aging in humans^3^. FGF23-deficient mice have a similar phenotype^6^. Both mouse strains suffer from drastically elevated plasma levels of phosphate and calcitriol due to the primary renal effect of FGF23 and Klotho. Importantly, the premature aging of Klotho- or FGF23-deficient mice is also a direct or indirect consequence of the hyperphosphatemia of the mice since maintaining them on a low phosphate or low vitamin D diet normalizes their life span^7^.

A high plasma FGF23 level has been found in patients with various acute and chronic disorders including renal (acute kidney injury, chronic kidney disease), cardiovascular (coronary heart disease, myocardial infarction, atrial fibrillation), inflammatory, and metabolic diseases^8^. The role of FGF23 in chronic kidney disease is established best: Plasma FGF23 is elevated before a marked decrease of glomerular filtration rate (GFR), and it exhibits a strong positive correlation with mortality, hypertrophy of the left ventricle, and disease progression^9^. Therefore, it is presently being considered as a valuable disease biomarker. However, it is yet incompletely understood whether and to which extent FGF23 contributes to pathophysiological processes rather than merely indicating them. At least, FGF23 has been shown to induce hypertrophy of the left ventricle independently of Klotho^10^.

Recently, inflammation has been shown to be a major trigger of FGF23 formation^11–14^. In line with this, pro-inflammatory cytokines including TNFα induce FGF23 production^15^.

Metabolic syndrome is characterized by hypertension, glucose intolerance, dyslipidemia, as well as obesity, and affects millions of patients world-wide and represents a significant health burden particularly in industrialized countries^16^. Although the complex pathophysiological processes have not yet been uncovered completely, it is clear that an imbalance between caloric needs and intake is the predominant factor. In mice, a diet rich in fats (high-fat diet (HFD)) induces metabolic syndrome^17–19^. Low-grade inflammation associated with metabolic syndrome is relevant especially for the development of glucose intolerance^20^. In this respect, pro-inflammatory cytokines derived from adipose tissue or the liver are a major source of inflammation in metabolic syndrome. Among those cytokines, TNFα has been found to play a predominant role^21^. Interestingly, an energy-dense diet has recently been demonstrated to upregulate the production of FGF23 in rats^22^.

Here, we sought to define the role of metabolic syndrome-associated TNFα production in HFD-induced FGF23 formation.

## Materials and methods

### Animals and treatments

All animal experiments were conducted according to the German law for the welfare of animals and were approved by the authorities of the state of Saxony-Anhalt. Experiments were performed in TNFα-deficient (tnf^−/−^) mice (from The Jackson Laboratory, Sulzfeld, Germany; Stock No: 005540; the generation and genotyping is available on the website of The Jackson laboratory) and in age- and sex-matched wild-type mice (tnf^+/+^) fed a control diet (Ssniff, Soest, Germany; standard diet for maintenance V1534).

At the age of 8–10 weeks, the mice were fed a HFD containing 70% kcal from fat (Altromin, Lage, Germany; C1090-70) for 3 weeks, and the body weight was recorded weekly. The animals had free access to food and tap water. Serum was taken before and on the last day of the treatment. The exact number of mice and the number of replications is provided in the figure legends. For all animal experiments, no randomization was used, no blinding was done, and no statistical test was applied to estimate the sample size.

### Serum parameters

To obtain blood specimens, the animals were lightly anesthetized with ether, and blood was drawn into heparinized capillaries by puncturing the retro-orbital plexus. Since the entire procedure takes less than a minute, it is unlikely to have a significant impact on our study. Serum concentrations of intact FGF23 and calcitriol were determined by ELISA kits (Immutopics, San Clemente, CA, USA; IDS, Frankfurt am Main, Germany). Inorganic phosphate was measured by a photometric method (Biocon^®^ Diagnostik, Vöhl/Marienhagen, Germany).

### Tissue collection and quantification of liver and adipose tissue Tnfα mRNA expression

For the determination of *Tnf*α mRNA abundance, total RNA was extracted from the liver and gonadal adipose tissue using the peqGold Trifast™ reagent (Peqlab, Erlangen, Germany) according to the manufacturer’s protocol. The RNA integrity was assessed by agarose gel electrophoresis and the RNA purity by measurement of the optical density at 260 and 280 nm. Single-strand cDNA was synthesized from 1.2 μg of total RNA at 42 °C for 60 min by use of the RevertAid^TM^ M-MuLV Reverse Transcriptase (MBI Fermentas, St. Leon-Rot, Germany) and oligo dT18 primers (Eurofins MWG Operon, Ebersberg, Germany). The mRNA expression level was determined by real-time polymerase chain reaction (RT-PCR) with the Rotor-Gene 6000 system (Corbett Research, Mortlake, Australia) using 2 µl cDNA templates, SYBR^®^ Green I (Sigma-Aldrich, München, Germany), 1.25 U Taq DNA polymerase (Promega, Mannheim, Germany), 500 µM dNTP (Ares Bioscience, Köln, Germany), and 13.3 pmol of a primer pair specific for *Tnfα* (NM_013693.2; forward 5′-AGT CCG GGC AGG TCT ACT TT-3′, reverse 5′-GGT CAC TGT CCC AGC ATC TT-3′). The *Tnfα* expression was normalized to the housekeeping gene glyceraldehyde-3-phosphate dehydrogenase (*Gapdh*, forward 5′-AAC GAC CCC TTC ATT GAC-3′, reverse 5′-TCC ACG ACA TAC TCA GCA C-3′) (in liver) or *18S* (forward 5′-GGG AGC CTG AGA AAC GGC-3′, reverse 5′-GGG TCG GGA GTG GGT AAT TT-3′) (in adipose tissue) using the ΔΔCt method.

### Cell culture

Cell culture was performed as previously described^23^. Briefly, UMR106 rat osteosarcoma cells (ATCC, Manassas, VA, USA) were cultured in DMEM high glucose medium (Gibco, Grand Island, NY, USA) supplemented with 10% FCS (Gibco) and 100 U/ml penicillin/100 µg/ml streptomycin (Gibco) under standard culture conditions. After 24 h, the cells were treated with or without TNFα (Sigma-Aldrich) for different periods.

### Quantitative RT-PCR (qRT-PCR)

Total RNA was isolated from the cells using Trifast reagent (Peqlab) according to the manufacturer’s instructions. Messenger RNA was transcribed with GoScript™ Reverse Transcription System (Promega) using 1.2 μg of total RNA and random primers. For qRT-PCR analysis, the final volume of the qRT-PCR reaction mixture was 20 µl and contained: 2 µl cDNA, 0.5–1 µM of a primer pair specific for rat *Fgf23* (forward 5′-TAGAGCCTATTCAGACACTTC-3′, reverse 5′-CATCAGGGCACTGTAGATAG-3′) or the housekeeping gene TATA box-binding protein (*Tbp*, forward 5′-ACTCCTGCCACACCAGCC-3′, reverse 5′-GGTCAAGTTTACAGCCAAGATTCA-3′), 10 µl GoTaq® qPCR Master Mix (Promega), and sterile water up to 20 µl. PCR conditions were 95 °C for 3 min, followed by 40 cycles of 95 °C for 10 s, 57 °C for 30 s, and 72 °C for 30 s. Quantitative RT-PCR was performed on a Rotor-Gene Q (QIAGEN, Hilden, Germany).

### Statistics

Data are provided as means ± SEM, *n* represents the number of independent experiments or number of mice per group, respectively. All data were tested for significance using the tests indicated in the figure legends. For serum FGF23, normal distribution was assumed. The data meet the assumptions of the respective tests. Variance was similar between the groups apart from the data in Fig. 2B and Fig. 4E. Therefore, Welch’s correction was applied in these cases. Only results with *p* < 0.05 were considered statistically significant.

## Results

**Fig. 1:**
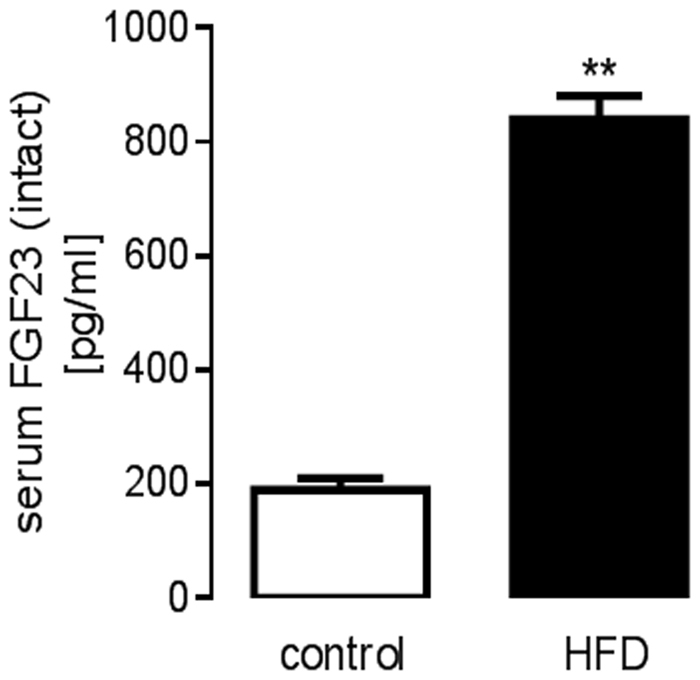
High-fat-diet-induced increase in the serum FGF23 concentration in tnf^+/+^ mice. Arithmetic means ± SEM of the serum intact FGF23 concentration (*n* = 3 mice, no replication) in tnf^+/+^ mice before (control) and after 3 weeks of feeding a high-fat diet (HFD); **p < 0.01 (paired, two-tailed *t*-test)

At the age of 8–10 weeks, we started to feed wild-type mice (tnf^+/+^) a HFD ad libitum for 3 weeks. Similar to what has recently been demonstrated in rats^22^, the HFD caused a strong increase (by almost four times) in the serum intact FGF23 level (Fig. 1).

**Fig. 2:**
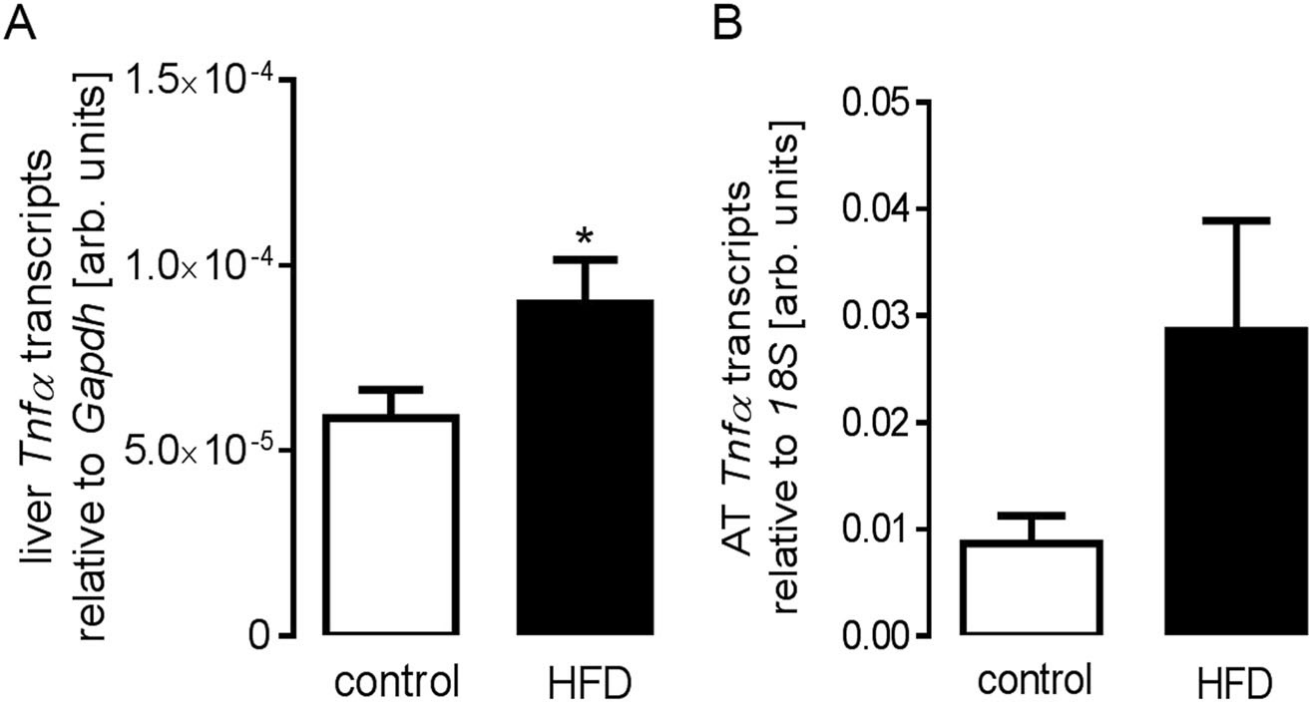
High-fat-diet-induced increase in liver *Tnfα* transcript levels in tnf^+/+^ mice. Arithmetic means ± SEM (*n* = 7 mice per group, one replication) of relative hepatic (**A**; unpaired, two-tailed *t*-test) and adipose tissue (AT). (**B**; unpaired, two-tailed *t*-test with Welch’s correction) *Tnfα* mRNA abundance (relative to *Gapdh* or *18S* mRNA) in tnf^+/+^ mice on control diet and on high-fat diet (HFD) for 3 weeks. *p < 0.05

HFD feeding and subsequent adipose tissue accumulation are associated with subclinical inflammation and the generation of the key pro-inflammatory cytokine TNFα. Hence, we found that feeding HFD indeed resulted in a significant increase in liver *Tnfα* mRNA expression levels (Fig. 2A) in tnf^+/+^ mice pointing to HFD-associated low-grade inflammation. Moreover, also adipose tissue *Tnfα* mRNA expression levels (Fig. 2B) tended to be higher in tnf^+/+^ mice on HFD, a difference, almost reaching statistical significance (*p* = 0.106).

**Fig. 3:**
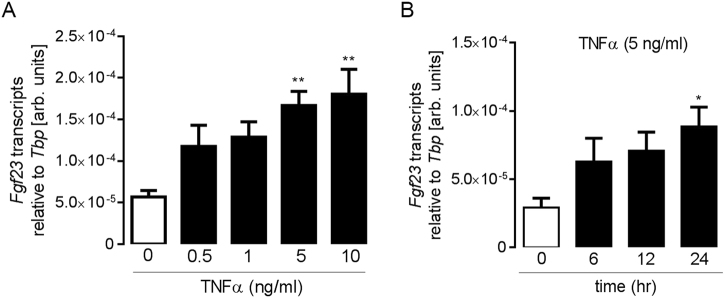
TNFα upregulated *Fgf23* transcripts in UMR106 cells. Arithmetic means ± SEM of relative *Fgf23* mRNA abundance (relative to *Tbp* mRNA) in UMR106 cells incubated for 24 h without (white bar) or with (black bars) TNFα at the indicated concentration (**A**; *n* = 5) or incubated with TNFα (5 ng/ml) for the indicated periods (**B**; *n* = 6); *p < 0.05 and **p < 0.01 (one-way ANOVA followed by Dunnett’s multiple comparisons test)

Next, we carried out cell culture experiments with UMR106 osteoblast-like cells to test whether TNFα is capable of stimulating FGF23 production as has been shown for IDG-SW3 cells^15^. According to Fig. 3a, a 24 h incubation with TNFα resulted in a dose-dependent upregulation of *Fgf23* mRNA transcript levels in UMR106 cells with significance at 5 and 10 ng/ml TNFα. The time dependence for the effect of 5 ng/ml TNFα is illustrated in Fig. 3B.

**Fig. 4:**
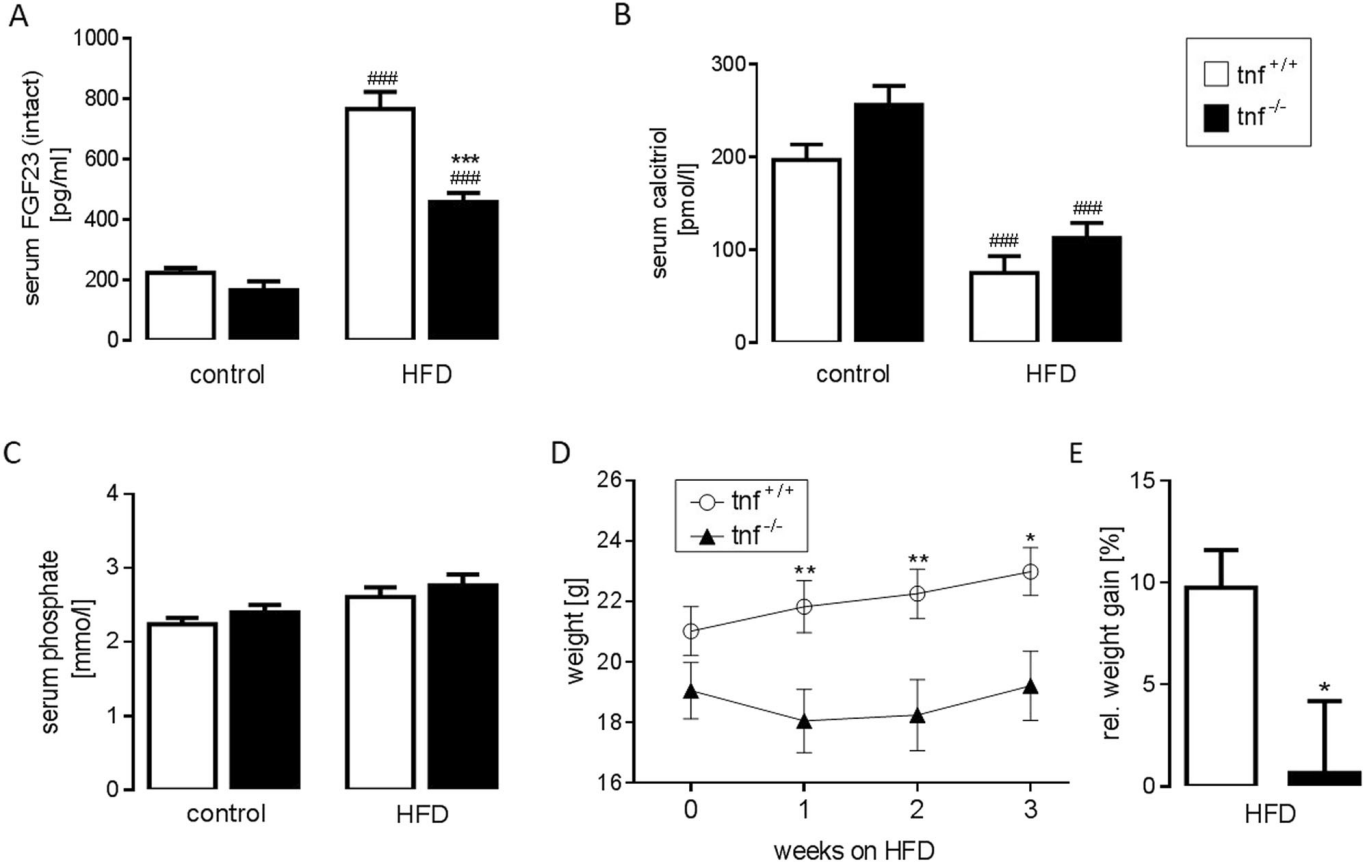
High-fat-diet-induced FGF23 production was blunted in TNFα-deficient (tnf^−/−^) mice. Arithmetic means ± SEM of the serum intact FGF23 concentration (**A**; *n* = 3 tnf^+/+^ mice and 7 tnf^−/−^ mice, one replication) and serum phosphate concentration (**C**; *n* = 11 tnf^+/+^ mice, and 8 tnf^−/−^ mice, two replications) in tnf^+/+^ mice (white bars) and tnf^−/−^ mice (black bars) before (control) and after 3 weeks of feeding a high-fat diet (HFD). Arithmetic means ± SEM of the serum calcitriol concentration (**B**; *n* = 8 mice per group) in a group of tnf^+/+^ mice (white bars) and tnf^−/−^ mice (black bars) on control diet or HFD fed for 3 weeks. Arithmetic means ± SEM (*n* = 13 tnf^+/+^ mice and 12 tnf^−/−^ mice, two replications) of the total body weight (**D**) in dependence of the duration of HFD and relative weight gain after 3 weeks of HFD (**E**); *p < 0.05, **p < 0.01, and ***p < 0.001 indicate significant difference between the genotypes; ^###^*p* < 0.001 indicates significant difference between control and HFD. (**A**–**C**: one-way ANOVA followed by Tukey’s multiple comparisons test; **D**: Holm-Sidak method; **E**: unpaired, two-tailed *t*-test with Welch’s correction)

Our last series of experiments explored whether the HFD-induced FGF23 production is dependent on TNFα formation. To this end, we compared tnf^+/+^ mice with tnf^−/−^ mice. On control diet, the serum intact FGF23 concentration was not significantly different between tnf^+/+^ mice and tnf^−/−^ mice (Fig. 4A). However, after 3 weeks of feeding the HFD, the serum intact FGF23 level was significantly different between the genotypes being nearly 50% lower in tnf^−/−^ mice compared to tnf^+/+^ mice (Fig. 4A). Serum calcitriol was not significantly different between tnf^−/−^ and tnf^+/+^ mice on either control or HFD, but was significantly lower in a group of HFD-fed mice compared to mice on control diet (Fig. 4B). Similarly, the serum phosphate concentration was not significantly affected by neither genotype nor diet (Fig. 4C). On control diet, no significant difference between the body weight of tnf^+/+^ mice and tnf^−/−^ mice could be observed (Fig. 4D). However, the HFD resulted in significantly stronger weight gain in tnf^+/+^ mice than in tnf^−/−^ mice (Fig. 4D, E).

## Discussion

According to our study, the stimulatory effect of a HFD on FGF23 formation was significantly blunted in gene-targeted mice devoid of pro-inflammatory TNFα (tnf^−/−^). This result suggests that a HFD stimulates FGF23 production in large part by inducing low-grade inflammation.

It is well established that energy-dense diets including a HFD favor the development of metabolic syndrome characterized by insulin resistance, dyslipidemia, obesity, and hypertension^24, 25^. This pathophysiological condition is associated with systemic low-grade inflammation^26^. In particular, a pivotal role for the pro-inflammatory cytokine TNFα in the development of obesity-induced insulin resistance has been demonstrated^21^.

Inflammation has emerged as a powerful factor driving FGF23 production^11^. Our study demonstrates that TNFα upregulated *Fgf23* transcript levels in UMR106 osteosarcoma-like cells. Importantly, TNFα is effective through transcription factor NF-κB^15^ and in line with this, NF-κB has also been demonstrated to enhance FGF23 synthesis^14^.

Elevated serum FGF23 concentrations are observed in acute and chronic renal, metabolic, and cardiovascular diseases^8^. Most of these clinical conditions are associated with inflammation. Therefore, similar to HFD feeding, these disorders may at least in part be effective in stimulating FGF23 production by enhancing the production of pro-inflammatory cytokines.

On control diet, the serum concentration of intact FGF23 was not significantly different between tnf^+/+^ mice and tnf^−/−^ mice although a tendency toward lower FGF23 in tnf^−/−^ mice was apparent. A pro-inflammatory milieu in HFD-treated animals, however, resulted in strong TNFα-dependent FGF23 generation.

Since an increase in serum FGF23 has been observed very early in some chronic disorders including chronic kidney disease, FGF23 has been suggested as a biomarker^27^. According to our results, an increase in serum FGF23 by almost four times was observed after 3 weeks of HFD feeding, a relatively short period as evident from a moderate increase in total body weight by only some 10% in tnf^+/+^ mice. Therefore, lower FGF23 may also indicate a better metabolic profile of an individual.

A major effect of FGF23 is the inhibition of renal calcitriol formation thereby lowering the serum calcitriol concentration^1^. Elevated FGF23 formation in HFD-fed mice could therefore be expected to decrease the serum calcitriol concentration. We did not, however, observe a significant difference in the serum calcitriol between the genotypes, although a tendency toward higher values in tnf^−/−^ mice was obvious.

Taken together, our study demonstrates that a HFD stimulates FGF23 production at least in part by inducing TNFα formation.
